# Measuring general mental health in early‐mid adolescence: A systematic meta‐review of content and psychometrics

**DOI:** 10.1002/jcv2.12125

**Published:** 2022-12-23

**Authors:** Louise Black, Margarita Panayiotou, Neil Humphrey

**Affiliations:** ^1^ The University of Manchester Manchester UK

**Keywords:** adolescence, measurement, mental health

## Abstract

**Background:**

Adolescent mental health is a major concern and brief general self‐report measures can facilitate insight into intervention response and epidemiology via large samples. However, measures' relative content and psychometrics are unclear.

**Method:**

A systematic search of systematic reviews was conducted to identify relevant measures. We searched PsycINFO, MEDLINE, EMBASE, COSMIN, Web of Science, and Google Scholar. Theoretical domains were described, and item content was coded and analysed, including via the Jaccard index to determine measure similarity. Psychometric properties were extracted and rated using the COSMIN system.

**Results:**

We identified 22 measures from 19 reviews, which considered general mental health (GMH) (positive and negative aspects together), life satisfaction, quality of life (mental health subscales only), symptoms, and wellbeing. Measures were often classified inconsistently within domains at the review level. Only 25 unique indicators were found and several indicators were found across the majority of measures and domains. Most measure pairs had low Jaccard indexes, but 6.06% of measure pairs had >50% similarity (most across two domains). Measures consistently tapped mostly emotional content but tended to show thematic heterogeneity (included more than one of emotional, cognitive, behavioural, physical and social themes). Psychometric quality was generally low.

**Conclusions:**

Brief adolescent GMH measures have not been developed to sufficient standards, likely limiting robust inferences. Researchers and practitioners should attend carefully to specific items included, particularly when deploying multiple measures. Key considerations, more promising measures, and future directions are highlighted.

*PROSPERO registration*: CRD42020184350 https://www.crd.york.ac.uk/prospero/display_record.php?ID=CRD42020184350.

## Graphical abstract

Mental health in adolescence is of key importance, given worsening trends and the fact that this is typically when psychiatric problems have first onset. To improve our understanding, brief general self–report measures are routinely used to document prevalence, monitor outcomes and evaluate interventions. This meta–review brought together measures from a range of approaches for the first time, assessing content and psychometrics. A wealth of conceptual and psychometric information is provided to help guide interpretation of studies using such measures and inform measure selection.

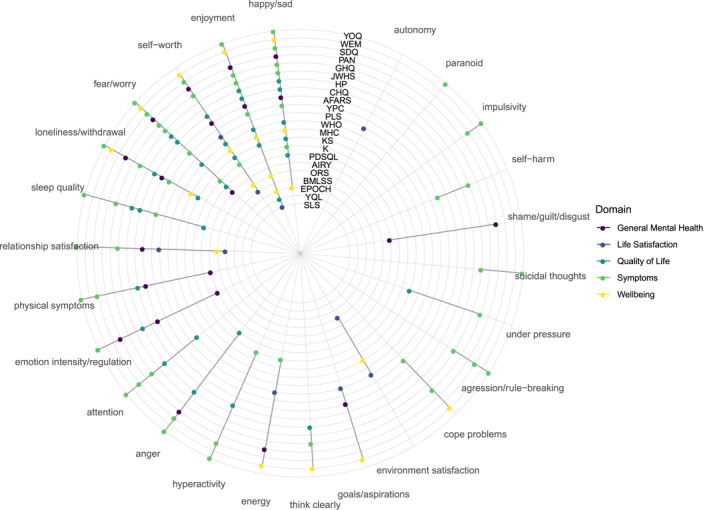

**Key points**

- Previous reviews of brief general mental health (GMH) measures have focused on psychometrics only, not integrated information across validation studies, and not extracted relevant subscales. We addressed these gaps, considering content and psychometrics together in the first meta‐review in this area.
- Three key findings suggest adolescent GMH suffers from poor conceptualisation: reviews classified measures into domains inconsistently; only a relatively limited range of experiences/question‐types were found across measures and domains; measures in the same domain tended not to be interchangeable in terms of content.
- Psychometric evidence was often lacking or poor.
- More psychometric/conceptualisation work, particularly consulting young people is needed.
- Researchers and practitioners should carefully evaluate item content and psychometric evidence before deploying brief adolescent GMH measures.

## INTRODUCTION

Accurate and efficient measurement of adolescent general mental health (GMH) are of vital importance: Adolescence, the phase starting around age 10 (Sawyer et al., 2018), appears pivotal for mental health problems, playing host to the first onset of the majority of lifetime cases (Jones, 2013). There is also evidence mental health of young people is worse than in previous generations (Collishaw, 2015). Despite a striking need to improve our understanding of mental health in this age group, research has typically faced major methodological problems, including low statistical power, poor measurement, and analytical flexibility (Rutter & Pickles, 2016). High‐quality research going forward will likely be underpinned by well‐developed brief general measures to facilitate large samples. Brief self‐report measures represent lower burden and are therefore more feasible when considering prevalence or response to intervention at appropriately large sample sizes (Humphrey & Wigelsworth, 2016). Specifically, time is a major concern for schools who are often called on to support administration of mental health questionnaires (Soneson et al., 2020), and in large panel studies (Rammstedt & Beierlein, 2014). Brief surveys are also recommended for work with adolescents to ensure better response rates (Omrani et al., 2018). This meta‐review focuses on the content and psychometric properties of self‐report measures to aid researchers and practitioners in selecting indicators and measures more likely to lead to valid inferences.

Various domains of GMH exist (e.g., disorders and wellbeing). However, it is currently unclear how these constructs relate to one another conceptually or their relative psychometric qualities. This is needed since some work has started to explore empirically the relationships between different domains (e.g., Black et al., 2019; Patalay & Fitzsimons, 2016), but findings in this area seem to be sensitive to measurement issues such as informant (Patalay & Fitzsimons, 2018) and operationalisation (Black et al., 2021). While a body of literature has been devoted to interpreting apparently paradoxical differences between positive and negative mental health outcomes (Iasiello & Agteren, 2020), we argue the known issues with adolescent mental health data (Bentley et al., 2019; Rutter & Pickles, 2016; Wolpert & Rutter, 2018) may mean such paradoxes are in fact artefacts, as some work has suggested (Furlong et al., 2017). Psychometric and conceptual properties must therefore be attended to going forward.

While analysis of item content is lacking, there is literature describing the theoretical domains to which measures belong. For instance, measures may be based on diagnostic systems such as the Diagnostic and Statistical Manual of Mental Disorders or frameworks such as hedonic, focused on happiness and pleasure, or eudaimonic, focused on broader fulfilment, wellbeing (Ryan & Deci, 2001). However, we chose to focus on item rather than construct mapping for several reasons: First, it is a known problem that measures with different labels sometimes measure the same construct (jangle fallacy), while others with the same label can measure different constructs (jingle fallacy; Marsh, 1994). Second, measures and their sub‐domains are often heterogeneous (Newson et al., 2020). Third, psychometric validations can be data‐driven, resulting in items with beneficial statistical properties prioritised over those considered to be theoretically key (Alexandrova & Haybron, 2016; Clifton, 2020). We therefore argue against further reification of construct boundaries.

From a policy perspective, there has often been a tendency to focus on diagnosis (Costello, 2015), while many have suggested attention is needed to a broader set of domains, particularly when considering early identification in general population samples (Bartels et al., 2013; Greenspoon & Saklofske, 2001; Iasiello & Agteren, 2020). Indeed, positive mental health is increasingly collected in large epidemiological studies (e.g., NHS Digital, 2018; Patalay & Fitzsimons, 2018). Given a need to answer the question of what should be considered under adolescent GMH, we did not seek an exhaustive definition prior to conducting our review (Black, Panayiotou, & Humphrey, 2020). Nevertheless, in the following paragraph we make explicit considerations that informed the meta‐review (see also eligibility criteria expanded in the Supporting Information).

Symptoms of mental ill‐health, but not individual disorders were considered relevant. We adopted this approach because of the need for brief general approaches, and consistent with previous reviews (Bentley et al., 2019; Deighton et al., 2014). Following these reviews of adolescent GMH, we also considered positive mental wellbeing, including affect via models such as subjective wellbeing, and quality of life. However, the aims and scope of our meta‐review, as well as issues raised in prior literature, meant it was important to impose some restrictions on wellbeing not included in these two prior reviews. The diffuse nature of eudaimonic wellbeing means it can be difficult to disentangle whether subdomains represent functioning or are predictors (Kashdan et al., 2008). Since there is a particular need to provide insight into measures for prevalence and response to intervention in adolescence, we argue non‐general domains of eudaimonic wellbeing such as perseverance, which might be a mechanism, should not be considered part of GMH. Similarly, though prior reviews have included entire quality of life measures, we felt it was important to consider only subdomains more clearly focused on mental health. These restrictions were also designed to keep the range of content relatively small so as not to artificially inflate the range of item content by including potentially proximal domains.

While adolescent GMH measure reviews have been conducted (Bentley et al., 2019; Deighton et al., 2014), these have not analysed content or provided robust psychometric ratings at the measure level. Furthermore, other measure reviews have often looked at narrower domains within GMH (e.g., Proctor et al., 2009), but this work across adolescent GMH has yet to be brought together. This is important since outcomes within GMH are sometimes referred to or treated interchangeably (Fuhrmann et al., 2021; Orben & Przybylski, 2019), and can be conceptually similar (Alexandrova & Haybron, 2016; Black et al., 2021). A meta‐review to consider conceptual and broader psychometric issues is therefore timely.

Furthermore, we argue the assessment of item content (e.g. the symptoms, thoughts, behaviours and experiences that are considered by measures) is a key omission. For instance, some researchers and practitioners may have clear theories about why one domain of GMH in particular is of interest (e.g., affected by an intervention). However, without explicit attention to content, results may be selected in a more data‐driven way. While it is the norm to register primary outcomes in trials, in adolescent mental health, some recommend multiple measures are explored for sensitivity (Horowitz & Garber, 2006). Observational studies also often collect multiple similar domains (e.g., NHS Digital, 2018). While such exploratory approaches play an important role, and flexibility can occur even after registration (Scheel et al., 2020), we suggest the content of measures should be attended to, particularly when combined. Before inferences are made about constructs, we must gain better understanding of how measures relate conceptually to increase transparency and validity.

Conceptual and psychometric insights are also vital given the recognised noisiness of adolescent mental health data (Wolpert & Rutter, 2018). Developmental considerations are particularly important when considering self‐report in this age range. For instance, issues such as inappropriate reporting time frames could introduce confusion (Bell, 2007; de Leeuw, 2011), or contribute to heterogeneity in assessments (Newson et al., 2020). Consider a case where a symptom measure (including e.g. depression) shows significant improvement after intervention but a wellbeing measure does not. If the wellbeing measure covers theoretically distinct content or the measures have differing reference periods, this is more likely to be a robust finding. However, if, for instance, both cover depression, affect or other indicators which could appear in either domain (Alexandrova & Haybron, 2016), this is less likely to be the case.

Another measurement issue which is gaining increased attention, but has yet to be considered for adolescent GMH, is the appropriateness of scoring mental health constructs by adding heterogeneous experiences together (Fried & Nesse, 2015). Since GMH is by definition likely to be broad, and measure developers can fall prey to data‐driven over conceptual considerations when selecting items (Alexandrova & Haybron, 2016; Clifton, 2020), it seems crucial to consider psychometric and conceptual issues together. In particular, the relative conceptual homogeneity within a measure might be considered useful context when assessing its statistical consistency.

The issues laid out above speak to contemporary debates: To aid comparison across studies there have been calls for common measures (Wolpert, 2020). However, a key problem is that different measures are likely appropriate for different contexts (Patalay & Fried, 2020). We argue the choice of measures for individual studies or to standardise across studies should be informed by analyses such as those reported here.

## METHOD

A systematic search was conducted to identify adolescent GMH measures following the preferred reporting items for systematic reviews and meta‐analyses (PRISMA) guidelines. We registered a number research questions. For clarity of reporting, these are grouped here into three overarching areas: theoretical domains, content analysis, and measurement properties.^1^ For theoretical domains we considered which were included in GMH in reviews. Based on our content analysis we considered the number of unique indicators; the presence of key common indicators across measures/domains; the proportions of items assessing broader themes by measure/domain (cognitive/affective/behavioural/physical, also coded at the item level); which measures best represented common indicators; and the similarity of measures within and between domains. For measurement properties we evaluated measures' time frames; psychometric properties; and statistical and conceptual consistency.

We defined several units of analysis. First, we use the term *theoretical domains* to refer to constructs described at the review level (e.g. life satisfaction). We grouped included reviews into theoretical domains inductively. Second, we use *indicator* to refer to specific question types capturing individual symptoms, thoughts, behaviours or experiences (e.g. sadness). Finally, we use *broad themes* to classify whether items tapped emotional, physical, social, cognitive or behavioural content.

Full search terms, eligibility criteria, inter‐rater reliability information, indicator codes, and *R* scripts are provided on the Open Science Framework (https://osf.io/k7qth/) and in the Supporting Information. The COnsensus‐based Standards for the selection of health Measurement INstruments (COSMIN) database of systematic reviews of measures was searched, as well as PsycINFO, MEDLINE, EMBASE, Web of Science, and Google Scholar. Reference lists of eligible studies were also searched. Search terms relating to the population (e.g., adolescen* OR youth*, etc.), measurement (e.g., survey* OR questionnaire*, etc.), and construct of interest (e.g., ‘mental health’ OR wellbeing, etc.) were combined using the AND operator. Where databases allowed, hits were limited to reviews, and English, since we aimed to review English‐language measures validated with English speakers.

To appraise the methodological quality of reviews from which we drew measures, we employed the quality assessment of systematic reviews of outcome measurement instruments tool (see Table S1 in the Supporting Information; Terwee et al., 2016). This provides a rubric for quality which covers aspects including clarity of aims, suitability of search strategy, and thoroughness of screening.

A subset of measures (drawn to be diverse in terms of domains and content) were initially discussed by all authors as the basis for the coding strategy. Two types of coding were performed, both at the item level, consistent with other work (Newson et al., 2020): indicator coding to record individual experiences such as happiness or anxiety, and broad theme coding, to capture whether items related to emotional, physical, social, cognitive or behavioural content. For the indicator coding, we aimed to code at a semantic level. However, given we could not be blind to the intended content of measures (e.g., measures' titles could give this away), coding could not be entirely inductive (Braun & Clarke, 2006). A hybrid approach allowed initial coding to be either specific or broad, with some codes collapsed into more general categories in subsequent coding, and others split up. After the initial meeting, the first author generated a full set of preliminary codes for all included items which were reviewed by the other authors. These were refined into a final set through discussion. In the final coding (https://osf.io/k7qth/), we aimed to collapse as much as possible without losing information. This was to avoid false positive differences between measures (Newson et al., 2020). Wherever possible, items were given a single indicator code, but for items assessing more than one experience (e.g. sadness and worry), two indicator codes were assigned.

Following Newson et al. (2020) each item was also assigned one or more broad themes (e.g., losing sleep over worry was considered physical and emotional). This allowed more conservative assessment of the similarity of measures. This was particularly important for our assessment of conceptual homogeneity. This approach is also consistent with much of the psychometric theory which typically underpins the measures we were interested in. This is namely that each item contributes information on the same state (Raykov & Marcoulides, 2011), making conceptual assessment of broader dimensions important alongside individual indicators.

As has been used elsewhere, similarity between measures was calculated via the Jaccard index (Fried, 2017). This index is the number of common indicators divided by the total number of indicators across a pair of measures, and thus reflects overlap from 0 (no overlap) to 1 (complete overlap). To calculate the index, each measure gains a 1 or 0 for presence or absence of a given indicator (regardless of frequency), making the index unweighted. This was desirable to avoid biased construct dissimilarity through our strategy of often including whole measures for domains like symptoms, but shorter subscales from quality of life. Items with double codes were both included as indicators for a given measure.

Though we initially intended to conduct secondary searches for psychometric evidence (Black, Panayiotou, & Humphrey, 2020), we instead opted to use primary psychometric studies of included measures cited in reviews. This was more feasible, was supported by the quality of reviews (which tended to use a range of databases, appropriate terms for measurement properties and clearly describe eligibility, see Table S1 in the Supporting Information), and frequent inclusion of measures in several reviews (see Figure 2). We reported only psychometric properties analysed in samples consistent with our criteria (e.g., not clinical samples or other age ranges), and included only studies reporting on relevant COSMIN elements at the level we considered (subscales or whole measures). All references and raw psychometric information extracted can be found at https://osf.io/k7qth/.

We used the COSMIN rating system for psychometric properties (Mokkink et al., 2018), which provides a standardised framework for grading the psychometric properties of measures in systematic reviews. It recommends consideration of content validity, structural validity, internal consistency, measurement invariance, reliability, measurement error, hypothesis testing for construct validity, responsiveness, and criterion validity. A few adaptations were necessary in the current study and are described in the Supporting Information. The rating takes the form: +, −, +/− (inconsistent), ? (indeterminate), and where no information was available we rated no evidence (NE).

In order to address statistical/conceptual consistency, we assessed whether measures/subscales were conceptually homogenous (H). We considered homogeneity to be present where only one broad theme was assessed. This was combined with statistical consistency (S), which we considered to be present where measures scored at least +/− for both structural validity and internal consistency. Measures could therefore be H+S+, H−S+, H+S−, or H−S−.

## RESULTS

### Review‐level results

**FIGURE 1:**
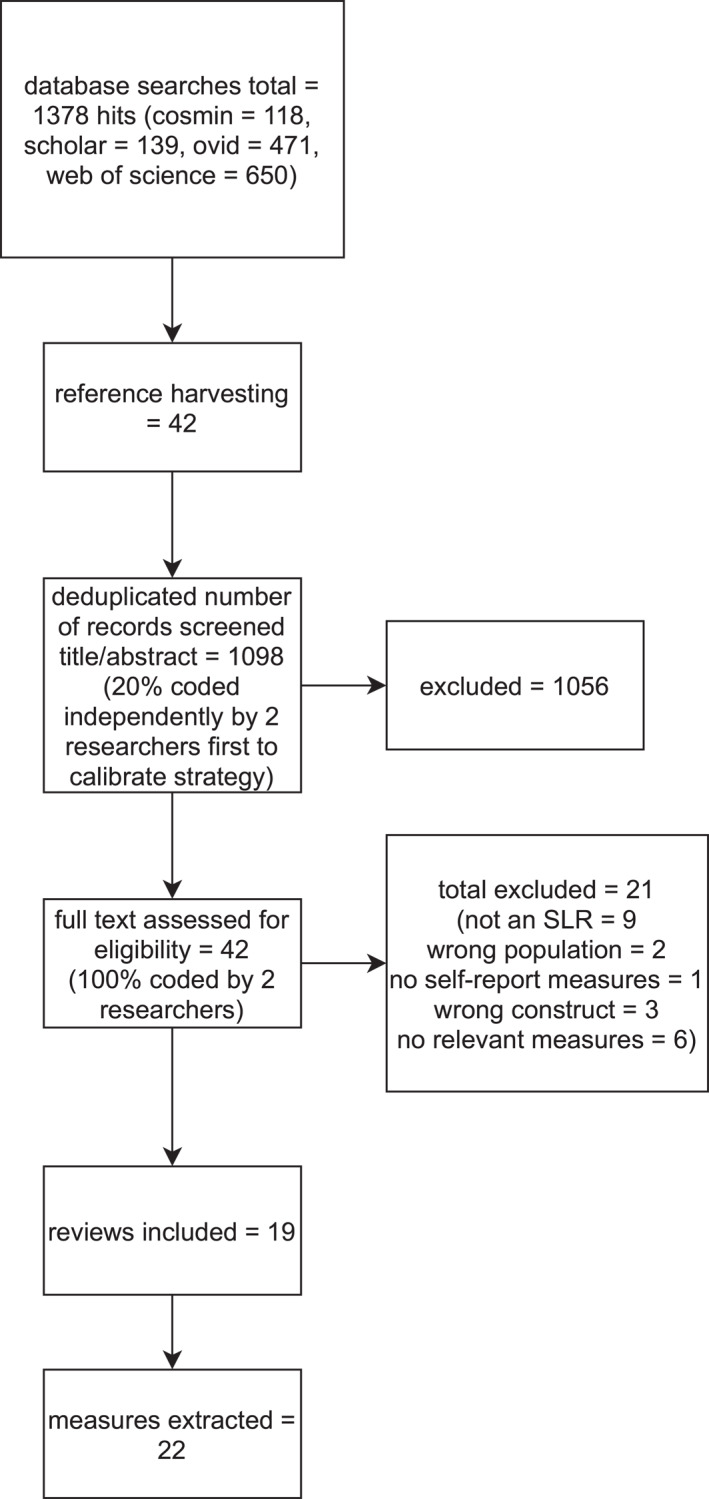
Flow diagram of review process.

A flowchart of the review stages is presented in Figure 1 with the primary reason for exclusion reported for full‐texts. The review resulted in the inclusion of 19 reviews and 22 measures (see also Table 1). The number of measures is after collapsing different versions of the same measure. We only extracted multiple versions where these were explicitly selected in reviews. The only measure for which multiple versions were reported on across reviews was KIDSCREEN, for which both the 52 and 27‐item versions were therefore extracted. We also did not count individual subscales separately. Therefore, while KIDSCREEN had multiple relevant versions and subscales, it was only counted as one measure (details of subscales can be found in Table 1).

**TABLE 1:** Overview of measures

Measure: subscale extracted where whole measure not included (acronym)	Number of items	Age range	Response format	Public domain/proprietary	Originally designed for adolescents	Time frame	Dimensions/subscales (not necessarily empirically validated)
General Health Questionnaire‐12 (GHQ‐12)	12	11–15	4‐Point	Proprietary	N	Recently	Unidimensional
Kidscreen‐52/27: psychological wellbeing, moods and emotions^3^ (KS)	6/7	8–18	5‐Point	Public domain	Y	Last week	Only psychological wellbeing and moods/emotions subscales extracted
Outcome Rating Scale (ORS)	4	13–17	Visual analogue	License required	N	Last week	Assumed unidimensional
Paediatric Quality of Life Inventory (PEDSQL)/PEDSQL‐short form: feelings	5/4	8–18	5‐Point	Free of charge for non‐funded academic research; proprietary for funded academic research; license for use required	Y	Past month	Only feelings subscale extracted
Strengths and Difficulties Questionnaire (SDQ)	15	11–18	3‐Point	Public domain‐ license for online use required	Y	Last 6 months	Only emotional symptoms, hyperactivity‐inattention, conduct problems subscales extracted
WHOQOL‐BREF: Psychological	6	13–19	5‐Point	Public domain	N	Last 2 weeks	Only psychological subscale extracted
Young Persons' Clinical Outcomes in Routine Evaluation (YP‐CORE)	10	11–16	5‐Point	Public domain	Y	Last week	Unidimensional
Youth Outcome Questionnaire (YOQ)	30	*M* = 11.93 (*SD* = 3.66)	5‐Point	Public domain‐ license required	Y	Last week	Scored as unidimensional
Kessler 6 (K6)	6	13–17	5‐Point	Public domain	N	Last 30 days	Unidimensional
EPOCH measure of adolescent well‐being: Happiness (EPOCH)	20	10–18	5‐Point	Free to use for non‐commercial purposes with developer acknowledgement	Y	None given‐ most items are present tense	Only happiness subscale extracted
Warwick‐Edinburgh Mental Wellbeing Scale (WEMWBS)	14	13–16	5‐Point	Free to use with developer permission	N	Last 2 weeks	Unidimensional
Child Health Questionnaire 87: mental health (CHQ)	16	10–18	4–6 point	Proprietary	Y	Past 4 weeks	Only mental health subscale extracted
Affect and Arousal Scale (AFARS)	27	8–18	4‐Point	Public domain	Y	Unclear	Positive affect, negative affect, physiological hyperarousal
Affect Intensity and Reactivity Measure Adapted for Youth (AIR‐Y)	27	10–17	6‐Point	Public domain	N	Unclear	Positive affect, negative reactivity, negative intensity
Positive and Negative Affect Schedule‐Child (PANAS‐C)	27	9–14	5‐Point	Public domain	N	Past few weeks or past 2 weeks	Positive affect, negative affect
Perceived Life Satisfaction Scale (PLSS)	19	9–19	6‐Point	Public domain	Y	Usually	Assumed unidimensional based on recommended scoring
Students' Life Satisfaction Scale (SLSS)	7	7–19	4‐Point	Public domain	Y	Past several weeks	Unidimensional
Brief Multidimensional Students' Life Satisfaction Scale (BMSLSS)	5	11–17	7‐Point	Public domain	Y	Unclear	Unidimensional
Juvenile Wellness and Health Survey 76: mental health problems (JWHS)	10	10–18	5‐Point	Public domain	Y	Unclear	Only mental health problems subscale extracted
Healthy Pathways self‐report: life satisfaction, emotional comfort, negative stress reaction (HP)	17	9–12	5‐Point	Public domain	Y	Variable: In the past 4 weeks/none	Only emotional comfort, negative stress reactions, life satisfaction subscales extracted
Mental Health Continuum Short‐Form: emotional wellbeing, psychological wellbeing (MHC‐SF)	7	12–18	6‐Point	Public domain	Y	Past month	Only emotional and psychological wellbeing subscales extracted
Youth Quality of Life Research Version: general QoL subscale (YQoL)	3	12–18	11‐Point	Public domain	Y	Unclear	Only general quality of life subscale extracted

Results of the quality assessment indicated mixed quality (see Table S1 in the Supporting Information). For instance, the vast majority of studies (94.74%) defined the construct of interest, and used multiple databases. However, reviewing, quality assessment and extraction of psychometric properties were often not clearly reported or were conducted only by a single researcher. Results are therefore in line with the general field of measure reviews (Terwee et al., 2016).

We included all criteria set out by Terwee et al. (2016). Since we had a specific age criterion, 100% of studies reported the population of interest. Nevertheless several reviews explicitly noted developmental considerations (Harding, 2001; Janssens, Thompson Coon, et al., 2015; Kwan & Rickwood, 2015; Rose et al., 2017), suggesting this had been considered in some detail.

**FIGURE 2:**
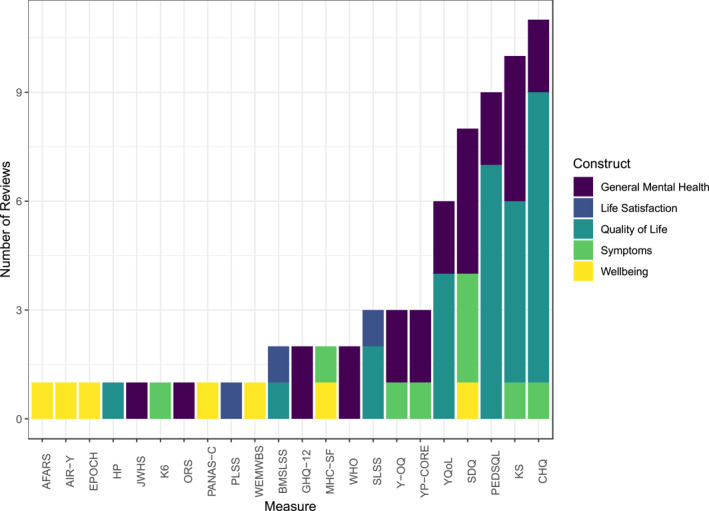
Summary of measures and reviews. Measures' full names can be found in Table 1.

**FIGURE 3:**
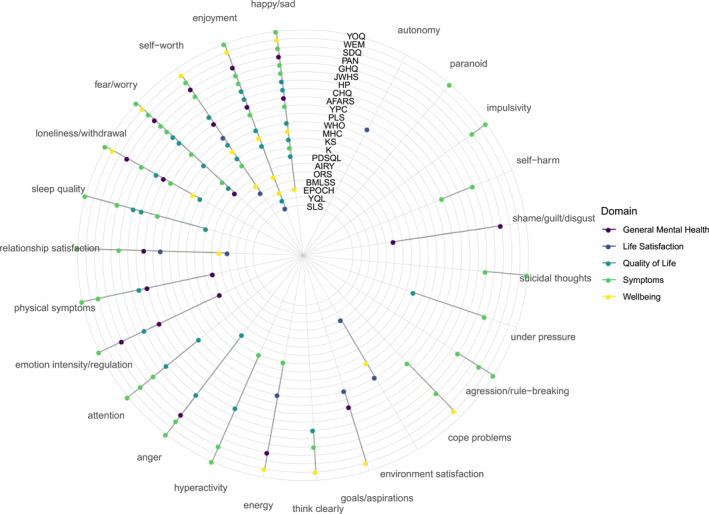
Twenty five indicators across measures by domain. Measures' full names can be found in Table 1.

The 19 reviews covered five theoretical domains (as described in reviews): GMH, holistic approaches including positive and social aspects (Bentley et al., 2019; Bradford & Rickwood, 2012; Kwan & Rickwood, 2015; Wolpert et al., 2008); symptoms (Becker‐Haimes et al., 2020; Deighton et al., 2014; Stevanovic et al., 2017); quality of life, including functional disability and patient‐reported outcome measures (Davis et al., 2006; Fayed et al., 2012; Harding, 2001; Janssens, Rogers, et al., 2015, Janssens, Thompson Coon, et al., 2015; Rajmil et al., 2004; Ravens‐Sieberer et al., 2006; Schmidt et al., 2002; Solans et al., 2008; Upton et al., 2008); wellbeing, positively‐framed strengths‐based measures or those with substantial proportions of positive items/subscales (Rose et al., 2017; Tsang et al., 2012); and life satisfaction (Proctor et al., 2009). Figure 2 demonstrates some measures appeared in several reviews under different domains (e.g., Child Health Questionnaire, CHQ and KIDSCREEN). Given the lack of consensus among reviews about which constructs measures fell under, we regrouped measures based on descriptions in validation papers cited in reviews. This resulted in the following domains which were used to inform subsequent analyses: affect, life satisfaction, quality of life, symptoms, and wellbeing (see also Figures 3 and 5 for this grouping).^2^

### Indicator and broad theme coding

The first round of item coding to describe items at the experience level (e.g., happiness) generated 45 codes which were then collapsed into a final set of 25 (see Figure 3). Since we had 285 items, the final reduction to indicators was substantial at 91.23%. The initial codes were typically more granular than the final set. For instance, aggression and rule‐breaking were initially each assigned a single code but these were combined in the final set (see https://osf.io/k7qth/ for the initial and final codes with descriptions). Similarly, the final emotion intensity/regulation code covered getting upset easily/impatience/strong positive and negative emotional responses/excited.

Five items had two codes applied resulting in three additional indicators being allocated to measures. The indicators in Figure 3 are ordered by how commonly they occur across measures, with happy/sad and enjoyment both occurring in 72.72% of measures, and autonomy and paranoid occurring across only 4.54%. The measures are ordered by number of indicators, with the outer‐most measure, YOQ, having the most, and SLS in the centre of the plot the least. Symptom measures covered the most indicators (84%), and life satisfaction measures the least (28%). The other domains each covered roughly half of all indicators.

**FIGURE 4:**
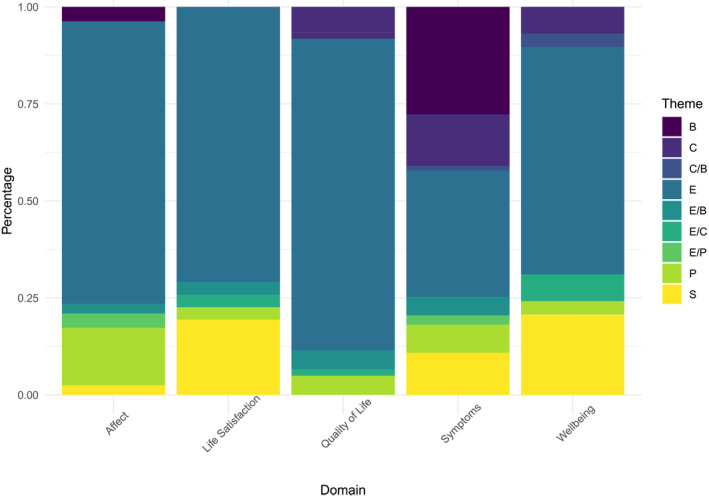
Broader themes across domains. B, behavioural; C, cognitive; E, emotional; P, physical; S, social.

Broader‐level themes (e.g., emotional or social content) are shown in Figure 4. These were not hierarchical but coded per item. Items within the same indicator often but not always had the same broad theme, reflecting our aim to collapse initial indicator codes as much as possible. For instance, 11 of the loneliness/withdrawal items were coded as tapping social content (e.g., ‘I withdraw from my family and friends’) while the remaining 5 were emotional (e.g., ‘feel lonely’). The majority of indicators tapped emotional experiences. Symptom measures had a higher proportion of behavioural and cognitive indicators, reflecting more coverage of externalising problems.

### Measure similarity

**FIGURE 5:**
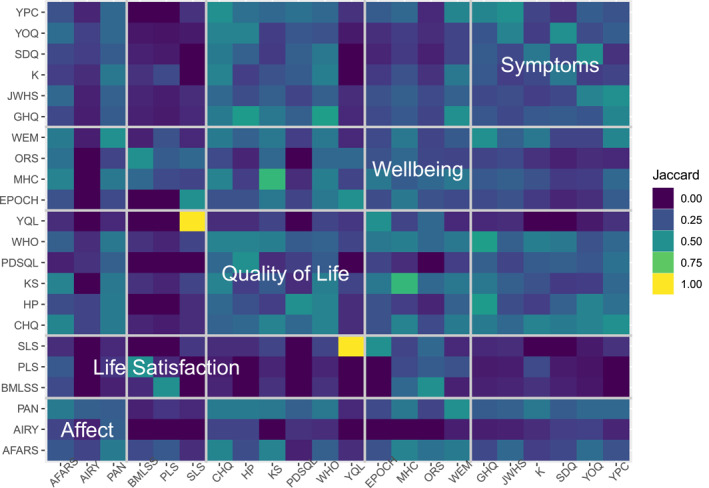
Jaccard index by measure. Measures' full names can be found in Table 1.

Jaccard overlap between pairs of measures covered the full range from 0 to 1 (*M* = 0.23, *SD* = 0.15). Only 14 (6.06%) measure pairs had similarity >0.50 (Figure 5). Of these, 10 (4.33% of all pairs) were for pairs of measures from *different* domains. Life satisfaction measures typically had low overlap with other measures. Affect measures also seemed to have relatively lower overlap while the remaining domains showed similar overlap. Average similarity for each measure with all others (shown on the diagonal of Figure 5) ranged from 0.09 (AIR‐Y) to 0.32 (CHQ), *M* = 0.23, *SD* = 0.06. Measures with higher average overlap were typically wellbeing and quality of life instruments. The pair of measures with perfect overlap (at this relatively broad indicator level, SLS and YQL), covered only enjoyment.

Measures appeared slightly more similar within than between domains. This can be seen by comparing the large diagonal boxes labelled with domain names in Figure 5 to other pairs in each row/column marked by pale grid lines (see also Table 2 for averaged Jaccard Index by domain).

**TABLE 2:** Average Jaccard indexes within (diagonal) and between (lower triangular) domains

	Affect	Life satisfaction	Quality of life	Wellbeing	Symptoms
Affect	0.33				
Life satisfaction	0.11	0.33			
Quality of life	0.24	0.13	0.42		
Wellbeing	0.24	0.24	0.30	0.38	
Symptoms	0.24	0.08	0.30	0.23	0.42

### Psychometric properties

The psychometric properties of measures are shown in Table 3. There was no evidence available for measurement error for any measure so this was omitted. Only six measures (27.27%) scored positively for content validity, a fundamental property (Mokkink et al., 2018). These measures all also scored favourably for construct validity, though no further positive results were found for these, suggesting overall low quality. This was echoed in mostly poor HS scores (i.e., a lack of support for conceptual and or conceptual consistency), which are shown in Table 3. For the 14 measures with clear time frames, all but one considered periods of 1–4 weeks (see Table 1).

**TABLE 3:** COSMIN ratings of measures

Measure domain	Measure (full name)	Content validity	Structural validity	Internal consistency	Reliability	Construct validity	Measurement invariance	Broad themes	HS score
Symptoms	GHQ‐12 (General Health Questionnaire)	−	+	+	NE	+	NE	4	H−S+
	SDQ (Strengths and Difficulties Questionnaire)	NE	+/−	−	?	−	−	Conduct = 2 Emotional = 3 Hyperactivity = 2 Total = 4	H−S−
	YP‐CORE (Young Person Clinical Outcomes in Routine Evaluation)	+	NE	?	?	+	NE	5	H−S−
	YOQ (Youth Outcome Questionnaire)	+	NE	?	?	+	NE	5	H−S−
	K (6) (Kessler)	−	NE	?	NE	−	NE	3	H−S−
	JWHS‐76 (Juvenile Wellness and Health Survey)	+	NE	?	NE	+	NE	4	H−S−
Quality of life	KS (KIDSCREEN)	+	+/−	?	−	+	+	Moods and emotions = 2 Psychological wellbeing = 2	H−S−
	PedsQL (Paediatric Quality of Life Inventory)	?	NE	?	NE	+	NE	2	H−S−
	CHQ (Child Health Questionnaire)	?	NE	?	NE	+	NE	3	H−S−
	YQoL‐R (Youth Quality of Life Research Version)	?	NE	?	+	+	NE	1	H+S−
	WHOQOL‐BREF (World Health Organization Quality of Life, Brief)	−	NE	?	NE	+	NE	2	H−S−
	HP (Healthy Pathways)	+	−	?	NE	+	+	Life satisfaction = 1 Emotional comfort = 1 Negative stress reaction = 2	H+S− / H−S−
Wellbeing	ORS (Outcome Rating Scale)	NE	NE	?	−	+	NE	2	H−S−
	EPOCH (Engagement, Perseverance, Optimism, Connectedness, Happiness)	NE	+/−	+	?	−	+	1	H+S+
	WEMWBS (Warwick‐Edinburgh Mental Wellbeing Scale)	−	+^a^	+	−	+	NE	5	H−S+^a^
	MHC‐SF (Mental Health Continuum Short‐Form)	NE	−	?	NE	+	NE	Emotional wellbeing = 1 Psychological wellbeing = 3	H+S− / H−S−
Affect	AFARS (Affect and Arousal Scale)	NE	+	+	?	−	NE	Negative affect = 1 Positive affect = 3 Physiological = 1	H+S+ / H−S+
	AIR‐Y (Affect Intensity and Reactivity Measure, Youth)	−	−	?	?	?	−	Positive affect = 3 Negative reactivity = 2 Negative intensity = 1	H−S− / H+S−
	PANAS‐C (Positive and Negative Affect Scale, Child)	+	NE	?	NE	+	NE	Positive affect = 3 Negative affect = 2	H−S−
Life satisfaction	PLSS (Perceived Life Satisfaction Scale)	NE	NE	?	NE	+	NE	4	H−S−
	SLSS (Student Life Satisfaction Scale)	NE	NE	?	?	+	NE	2	H−S−
	BMSLSS (Brief Multidimensional Student Life Satisfaction Scale)	NE	NE	?	?	+	NE	2	H−S−

Abbreviations: HS, conceptual Homogeneity Statistical consistency score; NE, no evidence.

^a^ Many residual correlations were added, likely driving up fit.

## DISCUSSION

This study systematically brought together measures across domains identified in systematic reviews as capturing adolescent GMH and is the first, to our knowledge, to consider content and psychometrics together. The current paper affords several new insights: First, theoretical domains were inconsistently defined, with individual measures frequently described as measuring different domains in different reviews. Second, despite a relatively large number of measures and domains, we found these to be captured by only 25 indicators, some of which appeared across the majority of measures/domains. Third, the narrow range of indicators was echoed in broader themes with most measures featuring emotional content. Despite this *quantitatively* narrow range of indicators, individual measures tended not to share the same subsets, and indicators were *qualitatively* diverse, reflecting the broad GMH construct expected. Fourth, quantitative analysis of measure overlap suggested only a few pairs of measures were highly similar, but these were largely for pairs from *different* domains. Together these first four insights suggest poor conceptualisation, with domains not clearly defined. Fifth, psychometric properties were typically not assessed or poor when considered at the measure level, suggesting insufficient development practices. Finally, though we considered only measures/subscales that were explicitly recommended for sum scoring, we found only a few with theme‐level homogeneity, and fewer still which also showed statistical coherence. This again suggests insufficient conceptual and psychometric development. When considering conceptual, psychometric properties and the interaction of these issues, our findings suggest brief adolescent GMH measures are not fit for purpose. Issues leading to this conclusion are further discussed below. Researchers and practitioners should therefore be cautious when selecting, analysing, and interpreting such measures, particularly if considering multiple outcomes. In the following sections we highlight particular considerations.

### Content analysis

A few indicators stood out as appearing in >50% of measures and 80%–100% domains: happy/sad, enjoyment, fear/worry, and self‐worth. This suggests these may be broadly useful, since validation processes have frequently led to their inclusion as indicators of GMH. Common indicators may also explain the classification inconsistency of measures into domains we found in reviews. While symptom measures had more idiosyncratic indicators, likely reflecting indicators that could only be framed negatively (e.g., suicidal thoughts), life satisfaction had the narrowest range of indicators. Despite this, some life satisfaction measures had relatively high thematic heterogeneity (see Table 3), likely reflecting that life satisfaction often considers satisfaction across a range of areas (e.g. social and emotional). Nevertheless, our findings suggest this breadth should not be considered reflective of GMH. The validity of findings (particularly external) aiming to capture GMH via life satisfaction may therefore be threatened.

The percentage reduction from items to indicators seen here, 91.23%, was greater than in similar studies of single disorders, which could be expected to be *more* homogenous than a broad construct such as GMH, where the number of items was reduced by 45.3%–77.3% (Chrobak et al., 2018; Fried, 2017; Hendriks et al., 2020; Visontay et al., 2019). The percentage reduction inevitably reflects how conservative coding was, though all studies described being cautious. We also saw the full range of overlap at the measure level whereas the aforementioned studies had smaller ranges (0.26–0.61). We found some pairs of measures (4.33%), from domains labelled as being different, had >50% overlap in terms of content, suggestive of the jangle fallacy. Generally though, similarity was low, even within domains, again suggesting domains are poorly defined. Researchers and practitioners should therefore attend to the specific items in questionnaires before deploying them, drawing on experts and analyses such as those presented here.

Not doing so could create problems with analysis and interpretation. For instance, even though we targeted subscales and measures designed to directly assess mental states, rather than antecedents, indicators of wider functioning were nevertheless included, such as relationships and aspirations. If GMH measures include such indicators, then careful treatment is needed when analysing potentially overlapping correlates. Relatedly, while some have called for measures of functioning (e.g., quality of life) to be consistently used to help compare studies (Mullarkey & Schleider, 2021), our analysis suggests that what constitutes mental health‐related quality of life or functioning is not consistently defined. These issues again speak to the fitness for purposes of adolescent GMH measures since it is clear researchers and practitioners cannot assume a given measure will precisely capture a well‐defined construct. In fact, this study suggests the opposite should be assumed, so that issues might be accommodated and reported transparently.

In terms of standardising measurement for capturing the range of GMH, no single measure or domain represented the entire spectrum. As discussed, we aimed to collapse codes wherever possible, emphasising the starkness of this finding. There were therefore no obvious candidates to be used as common metrics. The measures with the highest number of broad themes (see Table 3), also tended to have the most indicators (e.g., YOQ had the most with 15 while GHQ, WEMWBS, PANAS and SDQ all had nine, see Figure 3). However, these higher‐indicator measures were not interchangeable, with the greatest similarity between YOQ and SDQ at 50% (see Figure 5, code and data, https://osf.io/k7qth/). The inconsistency found at the review level is therefore reflected in our content findings. In terms of content, measures within theoretical domains are mostly not interchangeable, while some typically understood to capture different domains could be. This is of vital significance given the leap usually made from measure to construct when discussing findings, and makes clear potential problems of generalisability (Yarkoni, 2020). Again, we recommend researchers and practitioners assume measures and constructs are relatively unrefined and factor this into analysis and treatment decisions.

### Psychometric properties

Psychometric evidence was frequently lacking and COSMIN scores were low. Our results also confirm the general tendency to report only basic structural evidence (Flake et al., 2017). These findings highlight a lack of sufficient attention given to development practices in adolescent GMH. Though construct validity was frequently reported and positive, it should be treated with some caution since it has been suggested the type considered in the COSMIN rubric may not be valid if content and structural validity have not been considered (Flake et al., 2017), as was often the case here. In other words, without evidence that key stakeholders have been involved in developing the construct and or measure, and that items statistically cohere, the fact a given measure correlates with other similar outcomes is not very informative. Of the measures which scored positively for content validity, only KIDSCREEN and Healthy Pathways evaluated structural validity, scoring +/− and − respectively. Therefore, no measure benefited from both sound consultation work *and* had clear evidence that items successfully tapped a common construct.

Life satisfaction seemed particularly psychometrically problematic. Quality of life and outcome‐focused symptom measures, on the other hand, showed better content validity, that is, at a minimum involved young people at some stage of measure development. Given that our content analysis revealed measures labelled as the same domain were often not interchangeable, while those from separate ones could be, stake‐holder work on conceptualisation, and structural analysis to confirm and support this are arguably all the more necessary. Only measures' reference periods seemed to be relatively consistent and recent, in line with recommendations for this age group, though specific wording in individual measures could still introduce inconsistencies between measures or confusion (Bell, 2007).

### Conceptual and statistical coherence

As noted above, statistical coherence was typically unclear or poor. Similarly, though measures/subscales were recommended for sum scoring, they tended to cover more than one broad theme, suggesting conceptual unidimensionality was untenable. It is likely measures/constructs with thematic heterogeneity are not well suited to internal consistency metrics or sum scoring (Fried & Nesse, 2015). Similarly, reliability should only be prioritised by developers within theoretical units, since otherwise statistical reliability can be introduced via wording or other artefacts, rather than structural validity (Clifton, 2020).

Most measures covered more than one broad theme, and failed to meet our +/− COSMIN criterion for both structural validity and internal consistency (H−S−). We recommend such measures are not sum scored since this is not supported theoretically or statistically. Heterogeneous constructs may be desirable, particularly for GMH given one of its highlighted benefits is to provide broad insight (Deighton et al., 2014). We therefore question the (assumed) logic of total sum scores in this area. While items from measures included in this review could provide insight into GMH via methods other than sum scoring (e.g., network models or selecting particular items), further work is needed to validate such approaches.

GHQ‐12, WEMWBS and AFARS positive affect covered more than one broad theme but met our +/− COSMIN criterion for both structural validity and internal consistency (H−S+). This could be interpreted in several ways. It is possible these measures represent constructs that can be assessed from a variety of perspectives (indeed, positive affect was consistently heterogeneous). H−S+ could also signal data‐driven development without adequate consideration of whether sum scoring is theoretically appropriate. S+ could be the result of post‐hoc model modifications: In the case of WEMWBS, the addition of 28 error correlations in the adolescent validation (Clarke et al., 2011) is a potential cause for concern since it is unlikely these would be added if not needed to drive up model fit. Similarly, none of these three measures met our threshold for content validity with GHQ‐12 and WEMWBS both scoring poorly (−) as they were developed for adults. These considerations demonstrate the value of considering theoretical criteria alongside statistical properties. This is important, since the standard practice of basic statistical evidence (Flake et al., 2017) could miss critical insights. Our novel combined consideration of conceptual/statistical coherence offers a basis for doing so, which we hope others will develop further.

Various subscales, YQoL‐R, HP, AIR‐Y, covered only one broad theme, but failed to meet our +/− COSMIN criterion for both structural validity and internal consistency (H+S−). Unless other measures cannot provide relevant indicators, we suggest these should be treated with caution: They could have interpretability or other problems, since our conceptual analysis suggested conceptual issues were unlikely to be driving down statistical similarity between items. For instance, age appropriateness can be a particular concern and may negatively impact psychometric properties (Black, Mansfield, & Panayiotou, 2020).

Only EPOCH (happiness subscale) and AFARS (negative affect) covered only a single broad theme *and* met our +/− COSMIN criterion for both structural validity and internal consistency (H+S+). These subscales are likely more appropriate for sum scoring. However, the cost of this benefit is fewer GMH indicators (EPOCH contains four, and AFARS negative affect three). Again, this speaks to the lack of readiness of this field to land on common metrics. Additionally, these measures are by no means likely to be ideal in all scenarios. In particular, they are both potentially limited by not scoring positively for content validity. Our HS scoring system should therefore not be used to rank measures but be considered alongside issues such as indicators of interest and analytical approach.

### Strengths and limitations

This study systematically drew on a large body of systematic reviews, and therefore provides broad coverage of relevant measures and their properties. Novel conceptual and psychometric insights are provided through this approach. While some work has provided robust psychometric evaluation (Bentley et al., 2019), this was at the study level, while we were able to combine studies to provide more comprehensive ratings at the scale level. We also went beyond previous work by considering in detail which elements of quality of life were relevant to GMH, rather than providing information at the measure level (i.e. general quality of life) as has been done previously (e.g., Deighton et al., 2014). We therefore provide novel insight into the specific conceptual overlap of quality of life subdomains with other domains of GMH, as well as which subscales can be extracted and scored.

The current study provides a wealth of information for researchers and practitioners. Given the scope of such a project, some compromises were made. First, we were unable to conduct secondary searches for validation studies and therefore relied on the quality of searches conducted in reviews. Since we did not conduct secondary searches ourselves, we cannot be certain relevant papers were not missed. However, our meta‐review strategy meant that measures were picked up in multiple reviews (see Figure 2). Similarly, since we brought together work from previous reviews, rather than conducting searches for measures, relevant measures/versions have inevitably been missed. For instance, we are aware that the shorter version of WEMWBS has undergone some validation with adolescents (McKay & Andretta, 2017), but this was not picked up in any review. Our list of measures should therefore not be considered exhaustive, but in light of the quality of reviews (see Table S1 in the Supporting Information). Second, we did not assess potential methodological bias in validation papers, but rather rated only psychometric quality, for feasibility. When selecting measures we recommend researchers and practitioners consider this aspect in more detail, and should attend to factors such as the similarity of the sample to their application. Third, our assessment of homogeneity was somewhat crude. However, we based this on broader themes rather than indicators to take into account relationships between indicators. Considering themes rather than indicators was therefore conservative and less likely to underestimate homogeneity and appropriateness for sum scoring.

### Conclusion and recommendations

Conceptualisation was found to be problematic in adolescent GMH since measures were inconsistently defined within domains, indicators were often found across many of these, measures within domains were often not more similar to each other than between domains, and appropriate consultation practices were often not conducted. While GMH covered a diverse set of indicators, a relatively small number of these described the items we studied. This relatively narrow range, compared to for example, depression measures (Fried, 2017), was also seen in measurement time frames and that most items considered emotional content, whereas work looking at disorder measures found greater heterogeneity for these aspects (Newson et al., 2020). This suggests it may be possible to assess self‐report GMH briefly, though the psychometric work (including conceptualisation) needed to underpin this is currently largely lacking. It also suggests a singular approach to GMH could be appropriate, given that attempts to create distinct subdomains have resulted in common indicators.

Most measures also lacked sufficient psychometric evidence, and no measure or domain represented the entire spectrum of indicators. These factors make selection and interpretation of measures challenging in research and clinical applications, and suggest the field is not ready for a common metric. Furthermore, the lack of clear conceptualisation combined with insufficient psychometric evidence suggests the risk of measurement artefacts in applied research and clinical outcome monitoring is high. This suggests critical interrogation of existing findings is needed, and that progress may be limited by the measurement landscape.

We recommend that where assessment of GMH is the goal, new measures be developed, or existing ones revised. Our review provides excellent ground work for this by identifying the range of indicators that are likely theoretically relevant. Such analysis has been used to develop general measures for adults (Newson & Thiagarajan, 2020). Our additional assessment of psychometric information, would allow future work to ‘open up’ the codes found in measures with better content validity. This would allow consideration of item types within indicators developed in consultation with stake‐holders. For instance, our happy/sad code appeared in most measures, but the particular operationalisation of this going forward should preference measures which showed some content validity evidence.

In terms of selecting domains, symptom measures captured a broader range likely because some symptoms do not have theoretical positive poles. Researchers and practitioners should therefore consider whether theoretical breadth is important, whether the individual items are of interest, and whether they wish to sum score (this could be problematic for diverse item sets). Our findings also underscore that a single measure cannot be selected to represent any domain conceptually (given inconsistency within these). However, in terms of psychometrics, the following measures had at least evidence of content and construct validity: YP‐CORE, JWHS‐76 and YOQ (symptoms), KIDSCREEN and Healthy Pathways (quality of life), and PANAS‐C (affect). It is difficult to determine the relative psychometric quality of wellbeing measures reviewed given the lack of content validity evidence, though EPOCH (happiness) may be promising, given its match between conceptual and statistical coherence. From a GMH perspective, we recommend life satisfaction measures are avoided as these are psychometrically the weakest and show poorer coverage of GMH indicators. We recommend researchers and practitioners considering measures we reviewed draw on our code and data to assess specific content and properties relative to their context. Finally, our analysis suggests that researchers should not combine measures from different domains without accounting for likely similarity, and acknowledging potential systematic overlap due to common content.

## AUTHOR CONTRIBUTIONS

**Louise Black**: Conceptualization, Data curation, Formal analysis, Methodology, Visualization, Writing – original draft. **Margarita Panayiotou**: Conceptualization, Formal analysis, Methodology, Supervision, Writing – review & editing: **Neil Humphrey**: Conceptualization, Formal analysis, Methodology, Supervision, Writing – review & editing.

## CONFLICT OF INTEREST

The authors have declared that they have no competing or potential conflicts of interest.

### OPEN RESEARCH BADGES

This article has been awarded Open Materials, Preregistered badges. All materials and data are publicly accessible via the Open Science Framework at https://osf.io/k7qth/, in the Supporting Information, and https://www.crd.york.ac.uk/prospero/display_record.php?RecordID=184350. Learn more about the Open Practices badges from the Center for Open Science: https://osf.io/tvyxz/wiki.

## ETHICAL CONSIDERATIONS

Not applicable to this study.

## Supporting information

Supporting Information S1

## Data Availability

Open materials relating to method/results of the review are provided in Supporting Information.

## ENDNOTES

^1^
For transparency these relate as follows to our registration, theoretical domains covers RQ1, content analysis covers RQ2‐6, and measurement properties covers RQ7‐9.

^2^
Though we treated these as a single group, quality of life measures were noted to include subscales for the following domains that met our criteria: symptoms (CHQ, KIDSCREEN and PedsQL), wellbeing (KIDSCREEN), life satisfaction (Healthy Pathways, HP and Youth Quality of Life, YQoL), and psychological quality of life (KIDSCREEN and WH‐QoL), which contained a mixture of positive and negative indicators.

^3^
The 27 item measure has a single psychological wellbeing subscale (7 items), all but one of which appear across two subscales in the 52‐item version labelled psychological wellbeing (6 items) and moods and emotions (7 items).
