# Supplementary material for: Measuring general mental health in early‐mid adolescence: A systematic meta‐review of content and psychometrics
Source: JCPP Adv. 2022 Dec 23;3(1):e12125. doi: 10.1002/jcv2.12125 (PMC10241476; doi:10.1002/jcv2.12125)
Supplement: Supplementary file 1 — Supporting Information S1 [file JCV2-3-e12125-s001.docx]

Supporting Information for **Measuring general mental health in early-mid adolescence: A systematic meta-review of content and psychometrics**

Louise Black, Margarita Panayiotou, Neil Humphrey

The University of Manchester

Table of Contents

[Eligibility 2](#_Toc120870733)

[Inter-rater reliability 3](#_Toc120870734)

[Adaptations to COSMIN ratings 3](#_Toc120870735)

[Table S1. Quality of included reviews 4](#_Toc120870736)

[Searches 8](#_Toc120870737)

[Included reviews 10](#_Toc120870738)

# Eligibility

Since we conducted a meta-review, our inclusion and exclusion criteria were at the review level. Relevant measures were then extracted from reviews meeting our criteria. However, since reviews were often broad (e.g., several constructs or informants), they could only be included if they contained relevant measures. To be included, studies had to be systematic reviews, defined as including at least one database search (Terwee et al., 2016), and include brief self-report quantitative measures or subscales validated with general population adolescents^[[1]](#footnote-1)^ (evidenced in at least one review). We defined brief measures as 30 items or less. This was somewhat arbitrary but was chosen to capture measures likely to be completed quickly, given our interest in large-scale data collection. For instance the 25-item Strengths and Difficulties Questionnaire is described as taking 5 minutes to complete (Deighton et al., 2014). We considered only the 10-16 age range, given cognitive and social factors mean adult measures may be appropriate for older adolescents (de Leeuw, 2011). Where age ranges were wider than 10-16, validation studies met our criteria when the mean age fell within this range.

Relevant constructs were considered to be: psychiatric symptoms, as long as these were not disorder-specific (to be included reviews had to include measures tapping at least two disorders together or non-disorder specific symptoms); positive wellbeing, including life satisfaction and domain-general eudaimonic measures but not discrete eudaimonic subdomains such as perseverance, given the difficulty in disentangling whether these represent functioning or are predictors (Kashdan, Biswas-Diener, & King, 2008); and, psychological subscales from quality of life measures as long as they fitted with either of the first two construct criteria (e.g. not depression specific). Social and emotional measures focusing on constructs such as prosocial behaviour and empathy were excluded. Where individual subscales rather than whole measures were extracted, these had to have psychometric evidence of reliability and/or validity suggesting they could be used as standalone measures or explicit discussion of scoring at this level. Where reviews, information on measures (needed to determine eligibility), validation studies, or measures themselves were unavailable, these were requested from authors.

# Inter-rater reliability

Title and abstract screening was conducted in two stages: First a random subset of 20% of hits were screened independently by two authors. This pilot resulted in substantial agreement (95.9%, α = .67) (Landis & Koch, 1977), with disagreement mostly relating to vague abstracts with one rater erring on the side of caution. For sensitivity, the latter approach was adopted for the remaining records which were screened by the first author. Other disagreements related to study populations. It was decided reviews would be excluded if they focussed on a specific population (e.g., adolescents in social care), even if reviewed measures turned out to be validated in general populations. Full-texts of those retained after the title/abstract stage were all reviewed by two authors with 100% agreement after discussion. Given the heterogeneity of information provided across reviews, extraction was conducted primarily by the first author with the other authors consulted regularly throughout.

# Adaptations to COSMIN ratings

First, we did not extract data for responsiveness since we excluded clinical populations. Second, as advocated by COSMIN, and upon careful consideration of the literature, criterion validity was considered under hypothesis testing for construct validity, since clear gold-standard measures are not available for all domains, limiting comparisons. Construct validity was assessed using COSMIN standards, with the review team determining reasonable hypotheses and only rated as positive where at least 75% of these were met. Third, content validity was rated via criteria set out by Terwee et al. (2007) which match the COSMIN format (see below) but are briefer. Finally, while item response theory analyses are required to meet cut-offs for *all* criteria for satisfactory structural validity, an either/or approach is suggested for confirmatory factor analysis (CFA) fit indices. However, disagreement in fit indices are considered to warrant further investigation (Crede & Harms, 2019; Lai & Green, 2016), suggesting the either/or approach could lead to bias. We therefore considered CFA fit to achieve good structural validity standards if all CFA fit index thresholds were met. Since we considered only general populations aged 10-16, the subgroup approach to explaining inconsistent findings was not applicable, and as recommended we therefore considered the majority of evidence in each case to support ratings.

# Table S1. Quality of included reviews

| Quality Aspects | | | % | Median | Min | Max |
| --- | --- | --- | --- | --- | --- | --- |
| research question includes: | construct of interest | | 94.74 |  |  |  |
|  | population of interest | | 100.00 |  |  |  |
|  | type of measurement of interest | | 73.68 |  |  |  |
|  | measurement properties of interest | | 57.89 |  |  |  |
|  | all available instruments included | | 52.63 |  |  |  |
|  | only instruments included with some psychometric evidence | | 47.37 |  |  |  |
| search strategy | described | | 94.74 |  |  |  |
|  | no search terms/validated filter for: | measurement properties | 31.58 |  |  |  |
|  |  | type of instrument | 57.89 |  |  |  |
|  | databases | number searched |  | 4 | 1 | 10 |
|  |  | at least 2 | 94.74 |  |  |  |
|  |  | MEDLINE/PubMed | 84.21^[[2]](#footnote-2)^ |  |  |  |
|  |  | EMBASE | 31.58 |  |  |  |
|  |  | Additional/other databases | 100.00 |  |  |  |
|  | reference checking used | | 52.63 |  |  |  |
|  | no time limits used or used with good justification | | 73.68 |  |  |  |
|  | no language restrictions used | | 31.58 |  |  |  |
| reviewing | inclusion/exclusion | criteria clearly described | 89.47 |  |  |  |
|  |  | reasons for exclusion clearly reported | 47.37 |  |  |  |
|  | abstract selection by 2+ reviewers | | |  |  |  |
|  |  | Yes | 21.05 |  |  |  |
|  |  | No | 10.53 |  |  |  |
|  |  | partial | 10.53 |  |  |  |
|  |  | unclear | 57.89 |  |  |  |
|  | full-text selection by 2+ reviewers | |  |  |  |  |
|  |  | Yes | 21.05 |  |  |  |
|  |  | no | 10.53 |  |  |  |
|  |  | unclear | 68.42 |  |  |  |
|  | abstract and full-text selection by 2+ reviewers | | | | | |
|  |  | yes | 10.53 |  |  |  |
|  |  | no | 10.53 |  |  |  |
|  |  | unclear | 78.95 |  |  |  |
|  | quality assessment | 2+ reviewers | |  |  |  |
|  |  | yes | 15.79 |  |  |  |
|  |  | no | 15.79 |  |  |  |
|  |  | unclear | 63.16 |  |  |  |
|  |  | partial | 5.26 |  |  |  |
|  |  | extraction of measurement properties 2+ reviewers | | | | |
|  |  | yes | 21.05 |  |  |  |
|  |  | no | 21.05 |  |  |  |
|  |  | unclear | 52.63 |  |  |  |
|  |  | partial | 5.26 |  |  |  |
|  |  | instrument quality (psychometrics) considered | 84.21 |  |  |  |
|  |  | instrument quality 2+ reviewers | |  |  |  |
|  |  | yes | 15.79 |  |  |  |
|  |  | no | 15.79 |  |  |  |
|  |  | unclear | 63.16 |  |  |  |
|  |  | partial | 5.26 |  |  |  |
| synthesis | multiple studies of same instrument combined (e.g., best evidence synthesis or pooling) | | |  |  |  |
|  |  | yes-clear how | 15.79 |  |  |  |
|  |  | yes-unclear how | 68.42 |  |  |  |
|  |  | no | 15.79 |  |  |  |
|  | information provided: | per measurement property | 68.42 |  |  |  |
|  |  | only at domain level (reliability, validity, responsiveness) | 5.26 |  |  |  |
|  |  | only at whole instrument level | 15.79 |  |  |  |
|  | recommendations | for best instrument made | 21.05 |  |  |  |
|  |  | If yes, 1 instrument per construct/more than 1 | | | | |
|  |  | 1 | 5.26 |  |  |  |
|  |  | more than 1 | 15.79 |  |  |  |
|  |  | NA | 78.95 |  |  |  |
|  | psychometrics | reported as raw data | | | | |
|  |  | yes | 36.84 |  |  |  |
|  |  | no | 42.11 |  |  |  |
|  |  | partly | 21.05 |  |  |  |
|  |  | number reported/considered |  | 3.5 | 0 | 11 |
| conflict of interest | Declared, including funding source (both to be present) | | 31.58 |  |  |  |
|  | one of the authors also developer of included instruments (based on reference list search) | | 31.58 |  |  |  |

# Searches

| Database | Date | Terms | Hits | Dupli-cates | final |
| --- | --- | --- | --- | --- | --- |
| Scholar | 14/07/2020 | intitle:"review" (intitle:questionnaire\|intitle:measure\|intitle:scale\|intitle:assessment) (adolescent\|child) (intitle:"mental health"\| intitle:wellbeing\|intitle:psychopathology \| intitle:"life satisfaction"\|intitle:psychiatric \| intitle:"quality of life") | 140 | 1 | 139 |
| Cosmin | 02/07/2020 | (adolescen* OR teen* OR youth* OR child* OR minor* OR "young people*" OR "young person*" OR student* OR pupil* OR pediatric* OR paediatric*) AND ("mental health" OR wellbeing OR well-being OR "well being" OR "life satisfaction" OR "quality of life" OR psychopathology OR "mental* disorder*" OR "mental* ill*" OR psychiatr* OR "psychological adjustment") | 119 | 0 | 119 |
| Web of science core collection | 02/07/2020 | (TS=(adolescen* or teen* or youth* or child* or minor* or 'young people*' or 'young person*' or student* or pupil* or p*ediatric*) AND TI=(Survey* or Questionnaire* or checklist* or "check list*" or "Rating Scale*" or "Patient Reported Outcome Measure*" or measure* or tool* or instrument* or scale* or inventor* Screen*OR assess*) AND TS=("mental health" or wellbeing or well-being or "well being" or "life satisfaction" or "quality of life" or psychopathology or "mental* disorder*" or “mental* ill*” or psychiatr* or “Psychological adjustment”)) *AND***LANGUAGE:** (English) *AND* **DOCUMENT TYPES:** (Review) | 651 | 0 | 651 |
| Ovid: embase, PsychInfo, ,edline  APA PsycInfo <1806 to July Week 1 2020>  Embase <1974 to 2020 July 10>  Ovid MEDLINE(R) and Epub Ahead of Print, In-Process & Other Non-Indexed Citations, Daily and Versions(R) <1946 to July 10, 2020> | 13/07/2020 | 1: (adolescen* or teen* or youth* or child* or minor* or 'young people*' or 'young person*' or student* or pupil* or p?ediatric*).mp. [mp=ti, ab, hw, tc, id, ot, tm, mh, tn, dm, mf, dv, kw, fx, dq, nm, kf, ox, px, rx, ui, sy]  2: (Survey* or Questionnaire* or checklist* or "check list*" or "Rating Scale*" or "Patient Reported Outcome Measure*" or measure* or tool* or instrument* or scale* or inventor* Screen*OR assess*).m_titl.  3: ("mental health" or wellbeing or well-being or "well being" or "life satisfaction" or "quality of life" or psychopathology or "mental* disorder*" or "mental* ill*" or psychiatr* or "Psychological adjustment").mp. [mp=ti, ab, hw, tc, id, ot, tm, mh, tn, dm, mf, dv, kw, fx, dq, nm, kf, ox, px, rx, ui, sy]  4: 1 and 2 and 3  5: limit 4 to English  6: limit 5 to "systematic review"*  7: remove duplicates from 6 | 482 | 10 | 472 |

*This was achieved via selecting the additional limits function>publication type>systematic review

# Included reviews

Bentley N, Hartley S, Bucci S. Systematic Review of Self-Report Measures of General Mental Health and Wellbeing in Adolescent Mental Health. Clinical Child and Family Psychology Review. 2019;22(2):225-52.

Deighton J, Croudace T, Fonagy P, Brown J, Patalay P, Wolpert M. Measuring mental health and wellbeing outcomes for children and adolescents to inform practice and policy: a review of child self-report measures. Child and Adolescent Psychiatry and Mental Health. 2014;8(1):14.

Janssens A, Thompson Coon J, Rogers M, Allen K, Green C, Jenkinson C, et al. A Systematic Review of Generic Multidimensional Patient-Reported Outcome Measures for Children, Part I: Descriptive Characteristics. Value in Health. 2015;18(2):315-33.

Kwan B, Rickwood DJ. A systematic review of mental health outcome measures for young people aged 12 to 25 years. BMC Psychiatry. 2015;15(1):279.

Rose T, Joe S, Williams A, Harris R, Betz G, Stewart-Brown S. Measuring Mental Wellbeing Among Adolescents: A Systematic Review of Instruments. Journal of Child and Family Studies. 2017;26(9):2349-62.

Harding L. Children's quality of life assessments: A review of generic and health related quality of life measures completed by children and adolescents. Clinical Psychology & Psychotherapy. 2001;8(2):79-96.

Fayed N, De Camargo OK, Kerr E, Rosenbaum P, Dubey A, Bostan C, et al. Generic patient-reported outcomes in child health research: a review of conceptual content using World Health Organization definitions. Developmental Medicine & Child Neurology. 2012;54(12):1085-95.

Rajmil L, Herdman M, Fernandez de Sanmamed M-J, Detmar S, Bruil J, Ravens-Sieberer U, et al. Generic health-related quality of life instruments in children and adolescents: a qualitative analysis of content. Journal of Adolescent Health. 2004;34(1):37-45.

Bradford S, Rickwood D. Psychosocial assessments for young people: a systematic review examining acceptability, disclosure and engagement, and predictive utility. Adolesc Health Med Ther. 2012;3:111-25.

Wolpert M, Aitken J, Syrad HMM, Saddington C, Trustam E, Bradley J, et al. Review and recommendations for national policy for England for the use of

mental health outcome measures with children and young people.; 2008.

Becker-Haimes EM, Tabachnick AR, Last BS, Stewart RE, Hasan-Granier A, Beidas RS. Evidence Base Update for Brief, Free, and Accessible Youth Mental Health Measures. Journal of Clinical Child & Adolescent Psychology. 2020;49(1):1-17.

Stevanovic D, Jafari P, Knez R, Franic T, Atilola O, Davidovic N, et al. Can we really use available scales for child and adolescent psychopathology across cultures? A systematic review of cross-cultural measurement invariance data. Transcultural Psychiatry. 2017;54(1):125-52.

Davis E, Waters E, Mackinnon A, Reddihough D, Graham HK, Mehmet-Radji O, et al. Paediatric quality of life instruments: a review of the impact of the conceptual framework on outcomes. Developmental Medicine & Child Neurology. 2006;48(4):311-8.

Janssens A, Rogers M, Thompson Coon J, Allen K, Green C, Jenkinson C, et al. A Systematic Review of Generic Multidimensional Patient-Reported Outcome Measures for Children, Part II: Evaluation of Psychometric Performance of English-Language Versions in a General Population. Value in Health. 2015;18(2):334-45.

Ravens-Sieberer U, Erhart M, Wille N, Wetzel R, Nickel J, Bullinger M. Generic Health-Related Quality-of-Life Assessment in Children and Adolescents. PharmacoEconomics. 2006;24(12):1199-220.

Schmidt LJ, Garratt AM, Fitzpatrick R. Child/parent-assessed population health outcome measures: a structured review. Child: Care, Health and Development. 2002;28(3):227-37.

Solans M, Pane S, Estrada M-D, Serra-Sutton V, Berra S, Herdman M, et al. Health-Related Quality of Life Measurement in Children and Adolescents: A Systematic Review of Generic and Disease-Specific Instruments. Value in Health. 2008;11(4):742-64.

Upton P, Lawford J, Eiser C. Parent–child agreement across child health-related quality of life instruments: a review of the literature. Quality of Life Research. 2008;17(6):895.

Tsang KLV, Wong PYH, Lo SK. Assessing psychosocial well-being of adolescents: a systematic review of measuring instruments. Child: Care, Health and Development. 2012;38(5):629-46.

Proctor C, Alex Linley P, Maltby J. Youth life satisfaction measures: a review. The Journal of Positive Psychology. 2009;4(2):128-44.

References

Crede, M., & Harms, P. (2019). Questionable research practices when using confirmatory factor analysis. *Journal of Managerial Psychology, 34*(1), 18-30. doi:10.1108/JMP-06-2018-0272

de Leeuw, E. D. (2011). *Improving data quality when surveying children and adolescents: Cognitive and social development and its role in questionnaire construction and pretesting.* Retrieved from Finland: <http://www.aka.fi/globalassets/awanhat/documents/tiedostot/lapset/presentations-of-the-annual-seminar-10-12-may-2011/surveying-children-and-adolescents_de-leeuw.pdf>

Deighton, J., Croudace, T., Fonagy, P., Brown, J., Patalay, P., & Wolpert, M. (2014). Measuring mental health and wellbeing outcomes for children and adolescents to inform practice and policy: a review of child self-report measures. *Child and Adolescent Psychiatry and Mental Health, 8*(1), 14. doi:10.1186/1753-2000-8-14

Kashdan, T. B., Biswas-Diener, R., & King, L. A. (2008). Reconsidering happiness: the costs of distinguishing between hedonics and eudaimonia. *The Journal of Positive Psychology, 3*(4), 219-233. doi:10.1080/17439760802303044

Lai, K., & Green, S. B. (2016). The Problem with Having Two Watches: Assessment of Fit When RMSEA and CFI Disagree. *Multivariate Behavioral Research, 51*(2-3), 220-239. doi:10.1080/00273171.2015.1134306

Landis, J. R., & Koch, G. G. (1977). The Measurement of Observer Agreement for Categorical Data. *Biometrics, 33*(1), 159-174. doi:10.2307/2529310

Schmidt, L. J., Garratt, A. M., & Fitzpatrick, R. (2002). Child/parent-assessed population health outcome measures: a structured review. *Child: Care, Health and Development, 28*(3), 227-237. doi:<https://doi.org/10.1046/j.1365-2214.2002.00266.x>

Terwee, C. B., Bot, S. D. M., de Boer, M. R., van der Windt, D. A. W. M., Knol, D. L., Dekker, J., . . . de Vet, H. C. W. (2007). Quality criteria were proposed for measurement properties of health status questionnaires. *Journal of Clinical Epidemiology, 60*(1), 34-42. doi:<https://doi.org/10.1016/j.jclinepi.2006.03.012>

Terwee, C. B., Prinsen, C. A. C., Ricci Garotti, M. G., Suman, A., de Vet, H. C. W., & Mokkink, L. B. (2016). The quality of systematic reviews of health-related outcome measurement instruments. *Quality of Life Research, 25*(4), 767-779. doi:10.1007/s11136-015-1122-4

1. These did not have to be representative, but did have to be clearly not clinical or a subgroup (e.g., inner-city samples of school children were included). [↑](#footnote-ref-1)
2. The following review used MELINE Express only

   (Schmidt, Garratt, & Fitzpatrick, 2002) [↑](#footnote-ref-2)
